# Spatial Distribution of Sand Fly Vectors and Eco-Epidemiology of Cutaneous Leishmaniasis Transmission in Colombia

**DOI:** 10.1371/journal.pone.0139391

**Published:** 2015-10-02

**Authors:** Cristina Ferro, Marla López, Patricia Fuya, Ligia Lugo, Juan Manuel Cordovez, Camila González

**Affiliations:** 1 Laboratorio de Entomología, Subdirección Red Nacional de Laboratorios, Instituto Nacional de Salud, Bogotá, Colombia; 2 Centro de Investigaciones en Microbiología y Parasitología Tropical (CIMPAT), Facultad de Ciencias, Universidad de los Andes, Bogotá, Colombia; 3 Biomac, Facultad de Ingeniería, Universidad de los Andes, Bogotá, Colombia; Centro de Pesquisa Rene Rachou/Fundação Oswaldo Cruz (Fiocruz-Minas), BRAZIL

## Abstract

**Background:**

*Leishmania* is transmitted by Phlebotominae insects that maintain the enzootic cycle by circulating between sylvatic and domestic mammals; humans enter the cycles as accidental hosts due to the vector’s search for blood source. In Colombia, leishmaniasis is an endemic disease and 95% of all cases are cutaneous (CL), these cases have been reported in several regions of the country where the intervention of sylvatic areas by the introduction of agriculture seem to have an impact on the rearrangement of new transmission cycles. Our study aimed to update vector species distribution in the country and to analyze the relationship between vectors’ distribution, climate, land use and CL prevalence.

**Methods:**

A database with geographic information was assembled, and ecological niche modeling was performed to explore the potential distribution of each of the 21 species of medical importance in Colombia, using thirteen bioclimatic variables, three topographic and three principal components derived from NDVI. Binary models for each species were obtained and related to both land use coverage, and a CL prevalence map with available epidemiological data. Finally, maps of species potential distribution were summed to define potential species richness in the country.

**Results:**

In total, 673 single records were obtained with *Lutzomyia gomezi*, *Lutzomyia longipalpis*, *Psychodopygus panamensis*, *Psathyromyia shannoni* and *Pintomyia evansi* the species with the highest number of records. Eighteen species had significant models, considering the area under the curve and the jackknife results: *L*. *gomezi* and *P*. *panamensis* had the widest potential distribution. All sand fly species except for *Nyssomyia antunesi* are mainly distributed in regions with rates of prevalence between 0.33 to 101.35 cases per 100,000 inhabitants and 76% of collection data points fall into transformed ecosystems.

**Discussion:**

Distribution ranges of sand flies with medical importance in Colombia correspond predominantly to disturbed areas, where the original land coverage is missing therefore increasing the domiciliation potential. We highlight the importance of the use of distribution maps as a tool for the development of strategies for prevention and control of diseases.

## Introduction

Leishmaniases are a group of diseases caused by some species of intracellular protozoan parasites from the genus *Leishmania* (Kinetoplastida: Trypanosomatidae) transmitted to humans by the bite of Phlebotominae (Diptera: Psychodidae) insects. The vector gets infected with *Leishmania* after ingesting blood from an infected mammalian reservoir, where the parasite develops making mammals responsible of maintaining idle the infection in a transmission focus [1, 2]. In humans, clinical manifestations of the disease can vary, including cutaneous, mucous or visceral leishmaniases depending on a series of factors like: the infecting parasite species, host’s immune response and biochemistry of the insect’s saliva when it bites [3].

These parasitic diseases are prevalent in different habitats, from tropical rainforests in Central and South America to arid and semi-arid regions in Occidental Asia and the Americas [4,5]. In each area, parasite transmission depends, among other things, on specific micro-climate conditions, which define temporal patterns of annual cyclic abundance of vectors. Each one of these vectors has specific intervals of transmission which reaches its maximum when its population presents a greater number of females with eggs ready to be deposited [6].

The leishmaniases constitute a group of emerging diseases, registered in 98 countries of the world on 5 continents with reported endemic transmission. In the Americas, the estimated annual incidence of cutaneous leishmaniasis ranges between 187,000 to 307,800 cases [4]. The recorded increment in the distribution and number of cases, is principally a response to environmental changes, either anthropogenic or natural, which favor the rise and establishment of vector species’ populations in proximity to human settlements, increasing the public health concern around the world [7,8,9,10].

The current situation of leishmaniases transmission in Colombia has not only shown an increase in the number of cases, but also an important variation in comparison to previous eco-epidemiological patterns in transmission cycles: insect vector species have shown an increase in their spatial distribution and habitat ranges, being collected close to human settlements, and new vector-parasite associations have been detected [6,11,12,13]. Transmission cycles are occurring in their original sylvatic ecosystems but also peri-urban (e.g. Flandes, Tolima Department) and urban (e.g. Amador Neighborhood, Cartagena, Bolivar Department) cases have been recorded by the National Institute of Health’s Laboratory of Entomology (Personal communication C. Ferro).

Paradigms of *Leishmania* transmission have been broken, suggesting the establishment of new transmission cycles. For example, Parasite species are showing an impressive plasticity to adapt to new vector species under changing or new environmental conditions, distant from the previously known enzootic cycles [6,14]. Some parasites such as *Leishmania* (Viannia) *guyanensis* have shown their potential to migrate from the Amazon and Orinoquia regions and generate transmission foci in different ecological regions such as the Andean valleys of Chaparral located above 1000 m.a.s.l. (Tolima department) and Sucre department in the Caribbean Coast [6,14,15]. In Chaparral, the vector incriminated in *L*. *guyanensis* transmission was *Lutzomyia longiflocosa*, a species belonging to the Verrucarum group and known to occur only in the sub-Andean region, very different from the previous known vector *N*. *umbratilis* [6]. Furthermore, in Colombia and other Latin-American countries, the potential role of humans as reservoirs is under evaluation [16].

**Fig 1:**
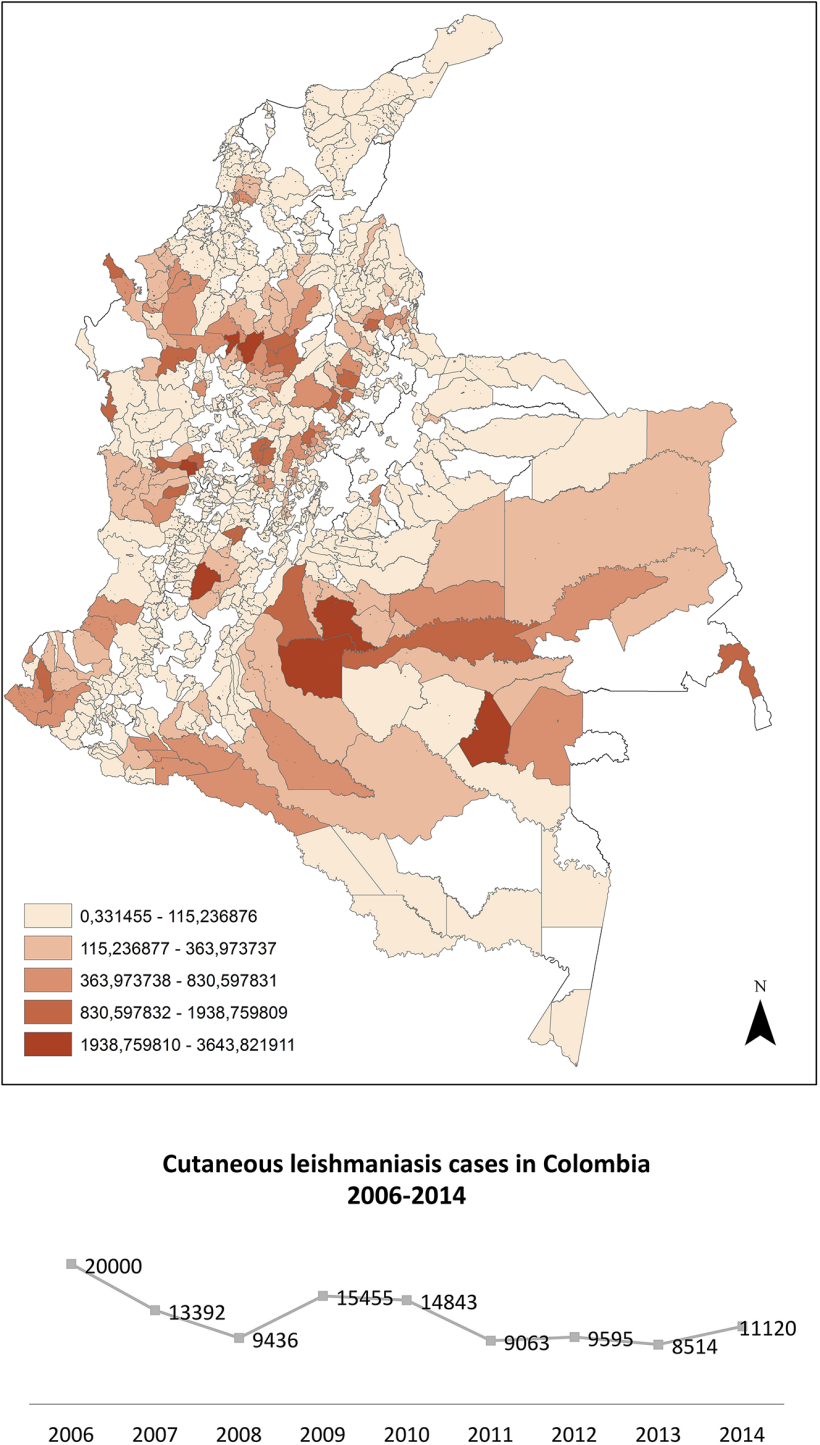
Cutaneous leishmaniasis prevalence. Data shown for each municipality from 2012 to 2014 and cases recorded by the National Institute of Health in Colombia between 2006 and 2014.

From the 163 species of Phlebotominae recorded in Colombia, only 14 have been proven as vectors; however the ecological complexity and the high biodiversity of the country allows to suspect that other species are or can act as vectors. The occurrence of leishmaniasis cases in Colombia constitutes an important Public Health problem. From 2006 to 2014, on average, 12,380 cases were recorded annually by the National Institute of health, showing inter-annual variation, possibly due to increases in rainfall patterns and the natural climatic variation of the disease (Fig 1)[17].

In spite of the high number of cases, a national strategy for leishmaniasis prevention and control has not yet been established and diagnosis and treatment still remain difficult. From this perspective, having a better understanding of variables involved in transmission such as the distribution of vector species’ and their relationship to environmental variables can be of great relevance for designing prevention and control strategies. The aim of this study was twofold: first, to update the known distribution of vector species responsible of cutaneous leishmaniasis using historical and current sand flies reports for Colombia; and second, to create potential distributions of the vectors using ecological niche modeling. The modeling allowed us to relate the distribution of insects to eco-epidemiological variables such as leishmaniasis’ prevalence, climate and land use. We postulate that this information constitutes an essential tool for health authorities because they can identify transmission foci and make inferences on potential vectors species.

## Methods

### Database assemblage and distribution maps

To identify vector species with medical importance for cutaneous leishmaniasis in Colombia, a search was performed to establish proven vector species (P) known to transmit *Leishmania* in the country and suspected vector species (S), found infected with *Leishmania* parasites either in Colombia or in other Latin-American countries. The list of proven and suspected species with the inclusion criteria is compiled in S1 Table.

A database containing taxonomical and geographic information was constructed, including the fields: genus and species (according to Galati’s nomenclature [18]) country, department, municipality, locality, longitude, latitude, collection date, and source. Data were obtained mainly from published literature [17,19,20,21,22,23] complemented with a literature search from 2000 to 2014 and using the search engine PubMed with the keywords "leishmaniasis Colombia", "*Lutzomyia* Colombia" and "Phlebotominae Colombia". For most data, geographic information was missing but collection sites were described so it was possible to spatially locate those sites using national and international gazetteers, Google Earth, Falling Rain (http://www.fallingrain.com/world/CO/), IGAC (http://www.igac.gov.co/igac). A further depuration process of the database was performed, revising that each locality actually coincided with the municipality and department described. When errors were found, records were either corrected, if allowed by the existing information, or discarded. Based on the final database, distribution maps were constructed for each of the 21 species and subspecies of medical importance.

### Climatic envelope and potential distribution

Climate envelope modeling was performed for each of the species included in the database by using the computer algorithm of maximum entropy Maxent. This free access program (http://www.cs.princeton.edu/~schapire/maxent/) calculates the probability of occurrence of species over each one of the cells in the country at 1km resolution. Thirteen bioclimatic variables were selected taking into account a correlation matrix, which evaluated variables that were both less correlated and had higher biological meaning for the studied species (Bioclimatic variables used: 1,2,4,8,9,10,11,12,15,16,17,18,19, variable explanation available at: http://www.worldclim.org/bioclim, last accessed 25 February 2015) [11,24]. Additionally 3 topographic variables were included: aspect, slope and elevation (available at: www.usgs.gov last accessed 25 February 2015) [11]. In order to reduce the bias presented in the extrapolation for the Worldclim coverages of the Orinoquia region, normalized differentiation vegetation indices of the region (NDVI) were obtained from the moderate resolution imaging spectroradiometer (MODIS) and principal components were composed. The first three PCA were selected (PCA1, PCA2, PCA3) which combined explained more than 60% of the variation and were included as another set of coverages together with the Worldclim models. The models were built with 75% training data and a 25% test data, the rest of the parameters were set to the default configuration chosen by the program. A binary output was selected in order to obtain presence/absence maps based on a 10 percentile training presence threshold. Therefore binary models of presence-absence were acquired for each one of the vector species involved.

Each one of the output models extracted from Maxent was evaluated and a selection criterion was established taking into account the area under the curve (AUC) displayed by the Sensibility vs Specificity table designed by the program with the purpose of excluding those deploying a bad prediction. The jackknife test obtained from Maxent allowed an analysis of the contribution of each environmental variable; in this way it was possible to determine the contribution of all variables to the distribution of each one of the species. Thereafter, distribution maps were created for each one of the selected vector species with the Arcmap 10.2 software. The known occurrence points for each one of the sand flies were overlaid on each of the corresponding distribution maps to characterize the contrast between the predicted and known presence zones and to identify model overprediction.

### Eco-epidemiological analyses

Eco-epidemiological analyses were performed to assess how vector’s predicted presence relate to leishmaniasis transmission areas and to land use coverages. This was done in order to investigate the potential for vector domiciliation and their implication in disease transmission in the Country.

Distribution of sand fly species in municipalities with recent cutaneous leishmaniasis cases was assessed to evaluate species than can be potentially acting as *Leishmania* vectors. Prevalence for each municipality was calculated using the cumulated number of cases from 2012 to 2014 [25] divided by the average total population of the municipality for the same period [26].

To assess species presence in transformed ecosystems, the percentage of presence of each species in those areas was established. Areas of species presence in transformed ecosystem were characterized to define the type of land use regarding the Etter map of general ecosystems of Colombia [27]. Vector potential areas of distribution were overlapped with Etter 1998 transformed ecosystem coverage in order to determine the associated percentage of known distribution of each species with agro-ecosystems and conserved areas, this in order to analyze potential for domiciliation processes and anthropogenic activities that most disrupt the species’ distribution.

To evaluate species richness, binomial maps of predicted potential distribution for all the species with significant models (AUC > 0.75) were summed. This map was overlapped to available epidemiological information to evaluate if areas with higher predicted species richness based on climatic envelope distribution concur with areas of higher number of cases as reported by the National Institute of Health.

## Results

### Spatial distribution of vector species

**Fig 2:**
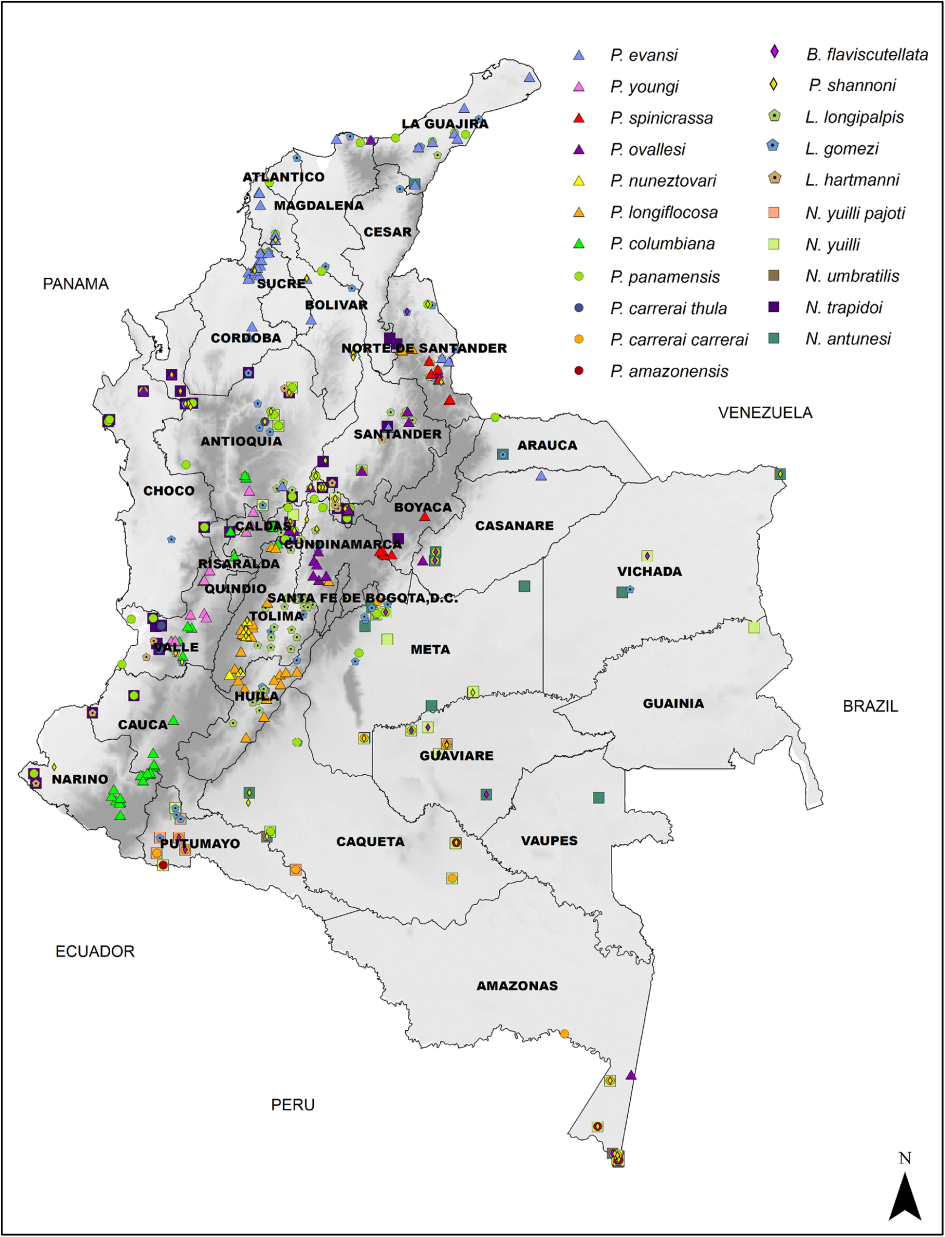
Distribution of 21 species of sand flies with medical importance in Colombia. The Department of Tolima has the largest number of collection records (n = 69) followed by Antioquia (n = 59), Boyacá (n = 53), Putumayo (n = 41) and Amazonas (n = 40). On the other hand, Vaupés (n = 1), Cesar (n = 1), Guainía (n = 2), Atlántico (n = 3), and Arauca (n = 4) displayed the least number of records, with Cesar and Vaupés presenting in total a single record each. San Andres and Providencia was the only department where no reports of any of the identified species of medical importance in the transmission of CL were collected. It is pertinent to mention that in the city capital, Bogotá, there is no presence of sand fly species and therefore it is currently a free risk zone for leishmaniasis although many imported cases are diagnosed and treated there.

In total, 673 records of unique localities of the 21 species and subspecies of medical importance included in the study were obtained, which were distributed in 30 of the 32 Departments of the Colombian territory as described next (Fig 2).

The species with the highest number of locality records were *Lutzomyia gomezi* (n = 98), *Lutzomyia longipalpis* (n = 56), *Psychodopygus panamensis* (n = 53), *Psathyromyia shannoni* (n = 52) and *Pintomyia evansi* (n = 46) (Fig 2, S1 Fig). The least number of locality records corresponded to the species *Psychodopygus carrerai thula* (n = 5), *Nyssomyia yuilli pajoti* (n = 7) and *Pintomyia nuneztovari* (n = 9) Moreover, *L*. *gomezi and P*. *panamensis* are the species with the widest distribution; they are observed in 24 and 23 of the Colombian Departments respectively (Fig 2, S1 Fig). *Lutzomyia gomezi* shows a wide distribution with records identified in low lands of the Departments of Valle del Cauca, Choco, La Guajira, Atlántico and Amazonas, and in high lands of the Departments of Antioquia, Norte de Santander, Caldas and Tolima, among others (Fig 2, S1 Fig). *Psychodopygus panamensis*, has also records in different altitudinal ranges, including lowlands and highlands of different Departments of the country which overlap with areas of *L*. *gomezi’s* distribution (Fig 2, S1 Fig). Even though these species have a wide distribution, their occurrence is majorly concentrated over the Caribbean, Pacific and Andean region with a few records in the Amazonian region. In contrast, *Pintomyia spinicrassa*, *Nyssomyia umbratilis*, *Pintomyia youngi*, *Psychodopygus carrerai thula and Nyssomyia yuilli pajoti* show a more restricted occurrence, only identified in less than four Departments of the country (Fig 2, S1 Fig). Overall, *Pintomyia columbiana*, *P*. *longiflocosa*, *P*.*nuneztovari*, *P*. *spinicrassa and P*. *youngi* have records dispersed exclusively over the Pacific and Andean regions, in contrast with *N*. *umbratilis* and *P*. *amazonensis* which are found exclusively in the Amazon region, principally in the lowlands of the Departments of Amazonas and Putumayo (Fig 2, S1 Fig).

In the Caribbean region, the species distributed are *L*. *gomezi*, *P*. *panamensis*, *P*. *shannoni*, *N*. *antunesi* and *P*. *ovallesi*. In this zone, occurrence records are limited to lowlands of the Departments of Bolivar, La Guajira and Magdalena (Fig 2, S1 Fig). On the other hand, a higher number of species was reported for the Orinoquia region: *N*. *antunesi*, *P*. *shannoni*, *B*. *flaviscutellata*, *L*.*gomezi*, *N*. *yuilli yuilli*, *P*. *carrerai carrerai*, *P*. *amazonensis*, *N*. *trapidoi* and *P*. *ovallesi*, principally in the departments of Meta and Vichada (Fig 2, S1 Fig). In the Amazon region these same species were collected, except *N*. *trapidoi*, and are predominantly distributed in the lowlands of Putumayo and Amazonas (Fig 2, S1 Fig). In the Andean and Pacific regions all of the 21 species and subspecies of medical importance were present. *Psychodopygus carrerai thula* distribution is restricted to the Pacific and Andean region and in spite of having a low number of occurrence records, is reported in high and low lands of the Departments of Antioquia, Valle del Cauca and Cauca (Fig 2, S1 Fig).

In the case of *L*. *longipalpis* and *P*. *evansi*, their distribution is principally along the Magdalena River Valley and the Caribbean Coast (Fig 2, S1 Fig). Overlapping distributions of these two species are observed in the Caribbean Coast and the lower Magdalena River Valley. *Lutzomyia longipalpis* is also present in the upper Magdalena River Valley, in the departments of Tolima, Huila and Cundinamarca.

### Climatic envelope and potential distribution

**Fig 3:**
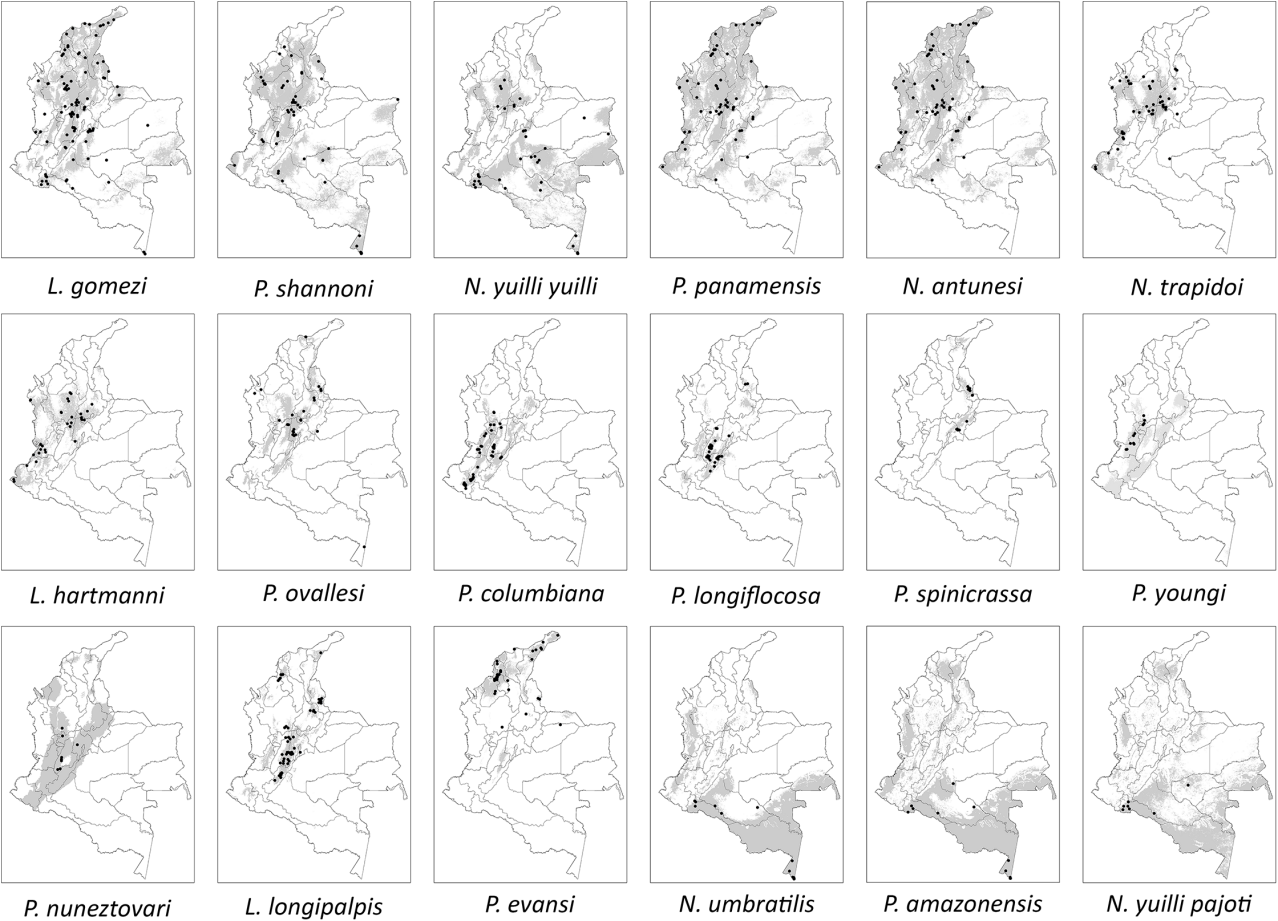
Binary maps showing predicted areas of presence (gray) and collection localities (dots) for each species.

Models with an AUC below 0.75 were discarded; therefore only 18 of the 21 species were adequately modeled and included for the predictive map construction (Table 1, Fig 3); *P*. *amazonensis* showed AUC of 0.77 but the p value was non-significant, so this species output should be carefully considered. Species with non-significant models were *B*. *flaviscutellata*, *P*. *carrerai carrerai*, *P*. *carrerai thula*.

**Table 1:** AUC and P values for each modeled species.

Species	Variable that produces the largest AUC when included	AUC value	Variable that produces the smallest AUC when omitted	AUC value
*B*. *flaviscutellata*	Slope	0.93	Mean Diurnal Range	0.39
*L*. *gomezi*	Mean Diurnal Range	0.72	Modis pca 1	0.81
*L*. *hartmanni*	Temperature Seasonality	0.86	Temperature Seasonality	0.92
*L*. *longipalpis*	Precipitation of Wettest Quarter	0.87	Precipitation of Warmest Quarter	0.96
*N*. *antunesi*	Mean Diurnal Range	0.75	Modis pca 1	0.82
*N*. *trapidoi*	Slope	0.79	Temperature Seasonality	0.75
*N*. *umbratilis*	Precipitation of Warmest Quarter	0.88	Mean Diurnal Range	0.92
*N*. *yuilli*	Annual Precipitation	0.72	Elevation	0.83
*N*. *yuilli pajoti*	Mean Diurnal Range	0.93	Mean Diurnal Range	0.93
*P*. *amazonensis*	Slope	0.68	Slope	0.70
*P*. *carrerai carrera*	Precipitation of Wettest Quarter	0.68	Temperature Seasonality	0.57
*P*. *carrerai thula*	Precipitation of Warmest Quarter	0.93	Precipitation of Wettest Quarter	0.65
*P*. *columbiana*	Mean Temperature of Driest Quarter	0.94	Precipitation Seasonality	0.97
*P*. *evansi*	Precipitation of Wettest Quarter	0.90	Precipitation of Coldest Quarter	0.87
*P*. *longiflocosa*	Precipitation Seasonality	0.91	Precipitation of Warmest Quarter	0.94
*P*. *nuneztovari*	Annual Mean Temperature	0.91	Elevation	0.89
*P*. *ovallesi*	Precipitation of Driest Quarter	0.86	Precipitation Seasonality	0.81
*P*. *panamensis*	Modis pca 2	0.71	Precipitation Seasonality	0.86
*P*. *shannoni*	Temperature Seasonality	0.84	Temperature Seasonality	0.77
*P*. *spinicrassa*	Slope	0.89	Annual Precipitation	0.98
*P*. *youngi*	Temperature Seasonality	0.96	Temperature Seasonality	0.95

Variables contributing the most according to Jackknife test performed by Maxent were: slope of the terrain, elevation, temperature seasonality and precipitation seasonality. On the contrary, variables contributing the least to species’ potential distribution were: mean temperature of wettest quarter and mean temperature of warmest quarter (Table 2).

**Table 2:** Jackknife AUC contributions of the environmental variables to each model.

Species	Variable that produces the largest AUC when included	AUC value	Variable that produces the smallest AUC when omitted	AUC value
*B*. *flaviscutellata*	Slope	0.93	Mean Diurnal Range	0.39
*P*. *evansi*	Precipitation of Wettest Quarter	0.90	Precipitation of Coldest Quarter	0.87
*L*. *gomezi*	Mean Diurnal Range	0.72	Modis pca 1	0.81
*L*. *hartmanni*	Temperature Seasonality	0.86	Temperature Seasonality	0.92
*L*. *longipalpis*	Precipitation of Wettest Quarter	0.87	Precipitation of Warmest Quarter	0.96
*N*. *antunesi*	Mean Diurnal Range	0.75	Modis pca 1	0.82
*N*. *trapidoi*	Slope	0.79	Temperature Seasonality	0.75
*N*. *umbratilis*	Precipitation of Warmest Quarter	0.88	Mean Diurnal Range	0.92
*N*. *yuilli*	Annual Precipitation	0.72	Elevation	0.83
*N*. *yuilli pajoti*	Mean Diurnal Range	0.93	Mean Diurnal Range	0.93
*P*. *amazonensis*	Slope	0.68	Slope	0.70
*P*. *carrerai carrerai*	Precipitation of Wettest Quarter	0.68	Temperature Seasonality	0.57
*P*. *carrerai thula*	Precipitation of Warmest Quarter	0.93	Precipitation of Wettest Quarter	0.65
*P*. *columbiana*	Mean Temperature of Driest Quarter	0.94	Precipitation Seasonality	0.97
*P*. *longiflocosa*	Precipitation Seasonality	0.91	Precipitation of Warmest Quarter	0.94
*P*. *nuneztovari*	Annual Mean Temperature	0.91	Elevation	0.89
*P*. *ovallesi*	Precipitation of Driest Quarter	0.86	Precipitation Seasonality	0.81
*P*. *panamensis*	Modis pca 2	0.71	Precipitation Seasonality	0.86
*P*. *shannoni*	Temperature Seasonality	0.84	Temperature Seasonality	0.77
*P*. *spinicrassa*	Slope	0.89	Annual Precipitation	0.98
*P*. *youngi*	Temperature Seasonality	0.96	Temperature Seasonality	0.95

*Psychodopygus panamensis (*300,699 km^2^ *)* and *P*. *amazonensis* (292,224 km^2^) are the species with the widest predicted distribution. *Psychodopygus panamensis* is found in 23 of the 32 departments of the country with records over all the regions of Colombia. In the case of *P*. *amazonensis’s* presence records are only observed in the Amazonia region, but some over predicted areas appear in the lowlands of the Pacific and Andean region. However due to geographic barriers, dispersal of this species is unlikely (Fig 3).

*Psathyromyia shannoni* (255,105 km^2^), *N*. *yuilli yuilli* (249,060 km^2^) and *L*. *gomezi* (245,581 km^2^), also possess large predicted potential distribution areas accordingly with their wide distribution. Even though *P*. *panamensis* is the species with the highest area of presence (300,699 km^2^), *L*. *gomezi* has a higher number of locality records as mentioned before. Overlapping distributions are shown for *P*. *shannoni*, *N*.*yuilli yuilli* and *L*. *gomezi* in the Andean and Pacific region but *N*. *yuilli yuilli* is not present in the northern part of the country (Fig 3).

*Pintomyia columbiana*, *P*. *longiflocosa*, *P*. *nuneztovari* and *P*. *youngi*, are also distributed over the Andean region with some of the records observed towards the Pacific region predicted as present in the departments of Cauca, Valle del Cauca and Nariño. *Pintomyia spinicrassa* is the species with the most confined predicted area of distribution occupying 19,093 km^2^. Its distribution is restricted to the northeastern part of the Andean Region (Fig 3).

*Lutzomyia longipalpis* and *P*. *evansi* are known vectors of Visceral leishmaniasis but have recently been classified as suspected vectors of CL. *Pintomyia evansi*’s distribution is limited to the northern side of the country, exhibiting a large predicted potential distribution in the northeastern part over the departments of Cordoba, Sucre and Bolivar; although this species show overprediction areas in the Magdalena river valley, its potential distribution is blocked by ecological barriers [11]. *Lutzomyia longipalpis*, on the other hand, is mainly distributed over the Andean region with the largest areas of occurrence over the departments of Huila, Tolima and Cundinamarca.

*Nyssomyia umbratilis* (237,592 km^2^) *and N*. *yuilli pajoti* (237,884 km^2^) exhibit similar spatial distribution; these species superimpose towards de southern and southwestern part of the Amazon region, particularly in the departments of Putumayo, Amazonas, Vaupés and Guainía. Over predicted areas are also observed in these species’ maps, as is the case of *P*. *amazonensis*. But even though suitable climatic characteristics may favor these species presence in the highlighted locations, geographic barriers also limit their distribution to the Amazon region (Fig 3).

Poorly sampled areas are in general located in the Amazonia and Orinoquia regions. Species predicted as present in those areas and having dispersal potential due to a continuous area of predicted distribution are: *N*. *umbratilis*, *P*. *amazonensis* and *N*. *yuilli pajoti*. These species also show overpredictions in the lowlands of the Pacific and Caribbean regions, with dispersal potential blocked by the Oriental Cordillera. Species with areas of over prediction in those regions include *P*. *panamensis*, *L*. *gomezi*, *P*. *shannoni* and *N*. *yuilli yuilli*. Some species show an interesting pattern of overprediction in the department of Guainia where a unique locality has ever been sampled according to our results (Fig 3). Interestingly, *P*. *nuneztovari* shows a greater distribution than already known (Fig 3).

### Eco-epidemiological analyses

#### Epidemiological data

Three species: N. trapidoi, N. antunesi and L. hartmanni, have more than 80% of its predicted distribution in areas with cases, while P. spinicrassa (57.6%) and P. youngi (61.36%) are the species with the smallest distribution in areas with reported cases. All sand fly species, except for N. antunesi are mainly distributed in regions with rates of prevalence between 0.33 to 101.35 cases per 100,000 inhabitants. Nyssomyia antunesi has 37.42% of its predicted presence in areas with prevalence rates between 102.53 and 405.26 and 27% in areas between 423.86 and 1071.31 cases per 100,000 inhabitants due to its presence in Meta, where the highest number of cases in the last years has been recorded.

*Nyssomyia trapidoi* (20.61%), and *L*. *hartmanni* (16.45%) predicted presence coincide with areas of prevalence rates between 423.86 and 1,071.31, possibly where active transmission foci are present.

Species distributed in areas with highest transmission rates are *N*. *antunesi* (5.25% of its distribution), *P*. *amazonensis* (3.40%), and *N*. *yuilli pajoti* (3.36%) although their areas of distribution in such regions are minimal (S2 Table).

#### Ecosystems

Regarding species distribution in Colombian Ecosystems, 76% of collection data points fall into transformed ecosystems of which 59% are rural areas with less than 20% of their original coverage; 23% are mixed agro ecosystems and 7% Coffee agro ecosystems. Species mainly distributed in transformed ecosystems and related to coffee plantations are *P*. *columbiana* (4.55% of collection localities and 15.58% of potential distribution), *P*. *youngi* (known 9.09%, potential 29.89%) *P*. *longiflocosa* (known 10%, potential 19.98%) and *P*. *nuneztovari* (knonw 11.11%, potential 31.09%).

Species associated with conserved areas both in their current and potential distribution are *P*. *amazonensis* (known 50%, potential 73%) *N*. *antunesi* (known 43.24%, potential 65.99%), *N*. *umbratilis* (known 53.85%, potential 80.4%) *N*. *yuilli yuilli* (known 41.03%, potential 63.53) and, *N*. *yuilli pajoti* (known 14.29%, potential 73.82%).

It is important to mention that some species had predicted presence in ecosystems where they have not yet been collected such as sugarcane, banana and oil palm plantations. *Pintomyia evansi*, *L*. *longipalpis*, *P*. *longiflocosa* and *P*. *ovallesi* were the species that brought out the highest predicted percentage of associated distribution with the sugarcane plantation agro-ecosystem, with values between 55% and 65%. The Cauca River Valley where this kind of crops is present appears as an area of potential distribution for *N*. *yuilli yuilli*, *P*. *ovallesi*, *L*. *longipalpis* and *P*. *evansi* possibly increasing this predicted relationship. The relevance of these predictions will be discussed.

#### Species richness

**Fig 4:**
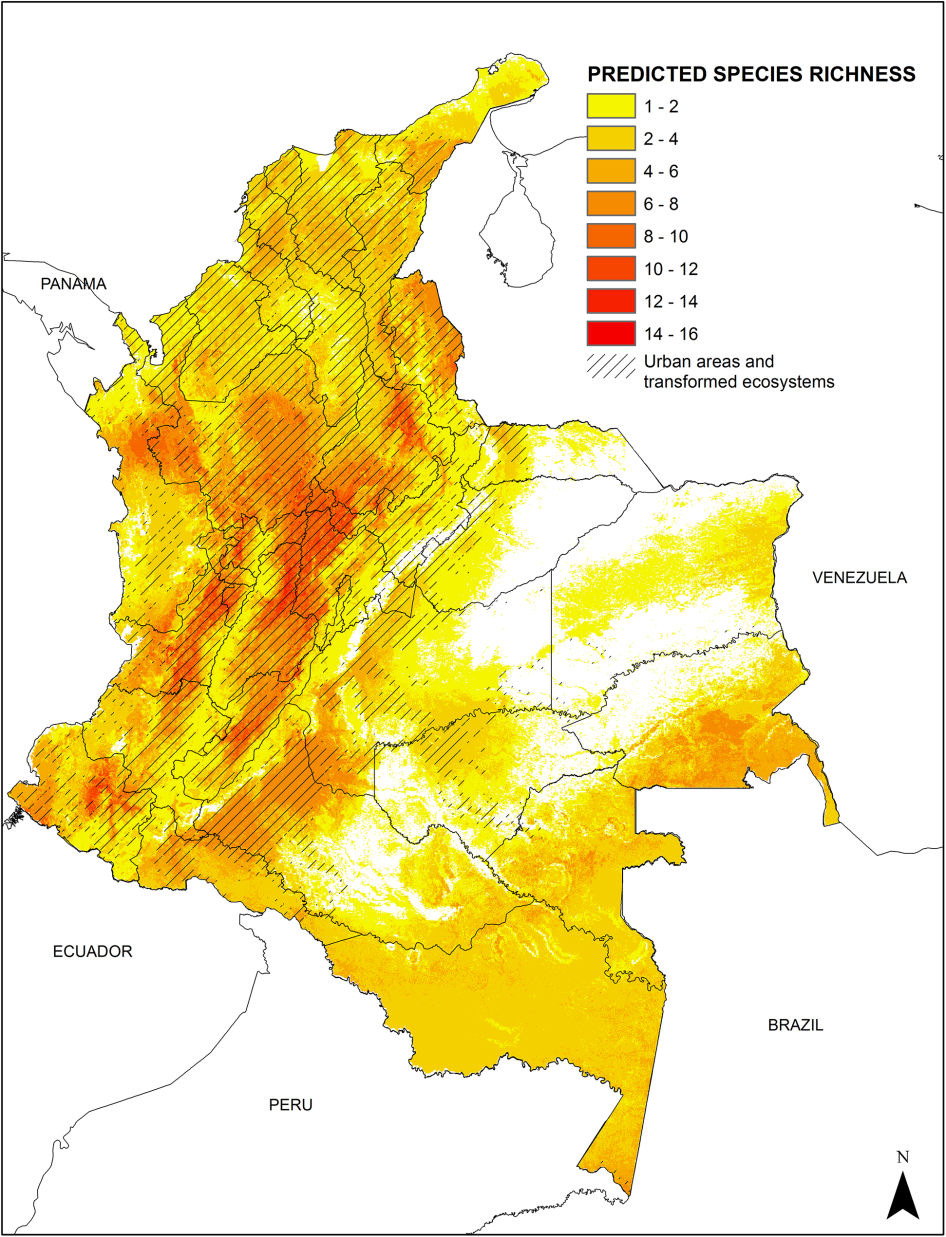
Species richness and transformed ecosystems. Regions with higher species richness in general coincide with areas of transformed ecosystems. In the Pacific Coast and the Amazonia regions, where conserved regions persist, high species richness is predicted. Species percent of predicted distribution in transformed ecosystems are as follows: *P*. *columbiana* (84.42%); *L*. *longipalpis* (83.76%), *P*. *longiflocosa* (79.98%) *P*. *ovallesi* (79.60%) *P*. *evansi* (74.41%), *P*. *spinicrassa* (72.56%), *P*. *youngi* (70.02%) *P*. *nuneztovari* (68.86%) *L*. *gomezi* (65.27%) *L*. *hartmanni* 62.60, *P*. *panamensis* 61.26, *N*. *trapidoi* 60.84, *P*. *shannoni* (57.05%), *N*. *yuilli yuilli* (36.19%), *N*. *antunesi* (33.86%), *P*. *amazonensis* (27%), *N*. *yuilli pajoti* (26%, *N*. *umbratilis* (19.39%).

Areas with higher predicted species richness based on climatic envelope distribution, are concentrated in the inter-Andean valleys, and particularly in the Magdalena River Valley. Areas with lower species richness are predicted in the Orinoquia region (Fig 4). Unfortunately, the available epidemiological information was not detailed enough to investigate a possible relation between species richness and leishmaniasis cases.

## Discussion

Vector species of cutaneous leishmaniasis have ample distribution in Colombia; their updated distribution proposed by this work will allow for identification of sand fly species with medical importance in each region, and its association with endemic transmission regions.

In general, known vector species tend to distribute in the Andean region, where a significant accumulation of collection records was observed, associated to areas of elevated leishmaniasis incidence over the years. Low numbers of collection records are observed in the Amazon, Caribbean and Orinoquia regions. This could be suggesting an inadequate entomological sampling related to the fact that perhaps most species are involved in sylvatic cycles and do not yet present a menace to the population. In a study conducted by Almeida *et al* (2015), the Amazon region appears as the second biome with the largest phlebotominae species richness, so it is mandatory to increase collections in that region in Colombia [28].

All sand fly species except for *N*. *antunesi* are primarily distributed in regions with low prevalence; possibly these are areas with endemic transmission where cases are constantly reported by health authorities but at low densities. Species present in areas with the highest transmission rates include *N*. *antunesi*, *P*. *amazonensis*, and *N*. *yuilli pajo*ti although their areas of distribution in such regions are reduced. Possibly these species are related to active transmission foci that have not been characterized yet.

The distribution maps obtained suggest a possible association between *L*. *gomezi* and *P*. *panamensis* within high-risk areas defined as endemic transmission zones in the departments of Tolima, Huila and Norte de Santander [29]. Other species such as *L*. *hartmanni*, *P*. *columbiana* and *P*. *longiflocosa*, were collected in a reduced set of localities, but their occurrence concur with areas that have high number of cases, suggesting high transmission rates associated to these species. Previous reports have incriminated these species as main vectors on several important CL foci in the country. For example, *L*. *hartmanni* was confirmed as the principal vector in the transmission of *P*. *colombiensis* in Otanche, Boyaca as well as in the department of Santander [30,31]. In addition, *P*. *longiflocosa* appeared to be the main vector in the domestic transmission of CL in the largest reported outbreak in Colombia that took place in Chaparral, Tolima in 2004 [6]. Also, it was postulated to be the vector responsible for the outbreak in Planadas, Tolima based on its abundance and ecological behavior [17]. Similarly, *P*. *columbiana* was identified as the most abundant species in the Samaniego, Santander focus due to its anthropophilic and zoophilic behavior as well as its ability to support full parasite development [32]

Interestingly, *P*. *nuneztovari* appears to have a wider distribution than was previously thought. This species has been associated with low abundance at the sites were it has been collected and thus is commonly considered to be a secondary vector. However its low abundance levels prevents it from maintaining a transmission cycle [15]

Our study includes only presence data so we are not able to make predictions on suspected vectors involved in transmission foci since local abundances must be assessed, however, it is known that the most abundant species is usually acting as the main vector in transmission foci [6,12,33].

Sand flies of medical importance in Colombia have distribution ranges that correspond predominantly to disturbed areas, mostly rural areas that have less than 20% of the original land cover. This situation has been demonstrated by other authors [34,35,36,37] that have shown that the appearance of new Leshmaniasis outbreaks could be related to the arrival of humans into regions where the sylvatic cycle is occurring [31,36,37]. In Colombia the presence of armed groups cause social instability that force displacement of large numbers of people into other regions, increasing the likelihood of infection because of exposure of susceptible populations to the insect vectors. *Lutzomyia gomezi* presented a high percentage of distribution in these ecosystems suggesting that this insect could be easily adapted to an anthropogenic-disturbed environment. Valderrama *et al*. 2014 [36] developed a study with *L*. *gomezi* in Panamá, in which she demonstrated that the wide distribution of the species is directly related with its high polymorphism caused by the high haplotipic diversity. This special feature is of great importance in terms of adaption and survival, mainly because it’s habitat has been constantly destructed by massive human migration [36].

Areas presenting the highest vector richness are associated with areas of high CL prevalence; these same sites also coincide with transformed ecosystems, which support the possibility of a domiciliation process. Likewise, we were able to identify poorly sampled sites, which require and additional effort in fieldwork for entomological captures and analysis. Unfortunately, available epidemiological information was not robust enough as to allow for further analysis. Information of cases at the municipality level is only available from 2012 to 2014 and cases at the department scale are too coarse as to establish any correlation. We were only capable of making a brief description of how each species with medical importance distributes in areas where cases are known to occur.

Finally, we highlight the importance of using high quality distribution maps as a tool for the development of strategies for prevention and control of infectious diseases. We are aware that many unpublished records could have improved this manuscript so we wish to promote and encourage data sharing and publication. However, the results obtained in this study are preliminary evidence of vector species distribution that support transmission cycles in different regions of Colombia.

## Supporting Information

S1 Fig Spatial distribution of sand fly species of medical importance by genus. The Andean Mountains are shown a) *Pintomyia* b) *Psychodopygus* c) *Lutzomyia* d) *Nyssomyia* e) *Bichromomyia* and *Psathyromyia* genus.
(PDF)

S1 Table List and incrimination status of all species included in the study. (PDF)

S2 Table Percent of area covered by potential distribution of each sand fly species in areas of cutaneous leishmaniasis by prevalence. (PDF)

## References

[1] Roque AL, Jansen AM. Wild and synanthropic reservoirs of *Leishmania* species in the Americas. Int J Parasitol Parasites Wildl. 2014 8 29;3(3):251–62. 10.1016/j.ijppaw.2014.08.004 25426421 PMC4241529

[2] Ashford RW. Leishmaniasis reservoirs and their significance in control. Clin Dermatol. 1996 Sep-Oct;14(5):523–32. Review. 8889331 10.1016/0738-081x(96)00041-7

[3] Davies CR, Reithinger R, Campbell-Lendrum D, Feliciangeli D, Borges R, Rodriguez N. The epidemiology and control of leishmaniasis in Andean countries. Cad Saude Publica. 2000 Oct-Dec;16(4):925–50. Review. 11175518 10.1590/s0102-311x2000000400013

[4] Alvar J, Vélez ID, Bern C, Herrero M, Desjeux P, Cano J, et al WHO Leishmaniasis Control Team. Leishmaniasis worldwide and global estimates of its incidence. PLoS One. 2012;7(5) 10.1371/journal.pone.0035671 22693548 PMC3365071

[5] Gramiccia M, Gradoni L. The current status of zoonotic leishmaniases and approaches to disease control. Int J Parasitol. 2005 10;35(11–12):1169–80. Review. 16162348 10.1016/j.ijpara.2005.07.001

[6] Ferro C, Marín D, Góngora R, Carrasquilla MC, Trujillo JE, Rueda NK, et al Phlebotominae vector ecology in the domestic transmission of American cutaneous leishmaniasis in Chaparral, Colombia. Am J Trop Med Hyg. 2011 11;85(5):847–56. 10.4269/ajtmh.2011.10-0560 Erratum in: Am J Trop Med Hyg. 2011. Dec;85(6):1154. 22049038 PMC3205630

[7] Isaza DM, Restrepo BN, Arboleda M, Casas E, Hinestroza H, Yurgaqui T. Leishmaniasis: knowledge and practice in populations of the Pacific coast of Colombia. Rev Panam Salud Publica. 1999 9;6(3):177–84. 10517095 10.1590/s1020-49891999000800005

[8] Ramírez JR, Agudelo S, Muskus C, Alzate JF, Berberich C, Barker D, et al Diagnosis of cutaneous leishmaniasis in Colombia: the sampling site within lesions influences the sensitivity of parasitologic diagnosis. J Clin Microbiol. 2000 10;38(10):3768–73. 11015400 10.1128/jcm.38.10.3768-3773.2000 PMC87473

[9] González C, Rebollar-Téllez EA, Ibáñez-Bernal S, Becker-Fauser I, Martínez-Meyer E, Peterson AT, et al Current knowledge of *Leishmania* vectors in Mexico: how geographic distributions of species relate to transmission areas. Am J Trop Med Hyg. 2011 11;85(5):839–46. 10.4269/ajtmh.2011.10-0452 22049037 PMC3205629

[10] OMS, miembros comite expertos. Control de las leishmaniasis: informe de una reunión del Comité de Expertos de la OMS sobre el Control de las Leishmaniasis, Ginebra, 22 a 26 de marzo de 2010.

[11] González C, Paz A, Ferro C. Predicted altitudinal shifts and reduced spatial distribution of *Leishmania infantum* vector species under climate change scenarios in Colombia. Acta Trop. 2014 1;129:83–90. 10.1016/j.actatropica.2013.08.014 23988300

[12] Vásquez Trujillo A, González Reina AE, Góngora Orjuela A, Prieto Suárez E, Palomares JE, Buitrago Alvarez LS. Seasonal variation and natural infection of *Lutzomyia antunesi* (Diptera: Psychodidae: Phlebotominae), an endemic species in the Orinoquia region of Colombia. Mem Inst Oswaldo Cruz. 2013 6;108(4):463–9. 10.1590/S0074-0276108042013011 23828011 PMC3970617

[13] Ocampo CB, Ferro MC, Cadena H, Gongora R, Pérez M, Valderrama-Ardila CH, et al Environmental factors associated with American cutaneous leishmaniasis in a new Andean focus in Colombia. Trop Med Int Health.2012 10;17(10):1309–17. 10.1111/j.1365-3156.2012.03065.x 22882595 PMC5079278

[14] Rodríguez-Barraquer I, Góngora R, Prager M, Pacheco R, Montero LM, Navas A, et al Etiologic agent of an epidemic of cutaneous leishmaniasis in Tolima, Colombia. Am J Trop Med Hyg. 2008 2;78(2):276–82. 18256429

[15] Bejarano EE, Uribe S, Rojas W, Velez ID. Phlebotomine sand flies(Diptera: Psychodidae) associated with the appearance of urban Leishmaniasis in the city of Sincelejo, Colombia. Mem Inst Oswaldo Cruz. 2002 7;97(5):645–7. 12219128

[16] Vergel C, Walker J, Saravia NG. Amplification of human DNA by primers targeted to *Leishmania* kinetoplast DNA and post-genome considerations in the detection of parasites by a polymerase chain reaction. Am J Trop Med Hyg. 2005 4;72(4):423–9. 15827280

[17] Cárdenas R, Romoz GM, Santamaría E, Bello F, Ferro C. *Lutzomyia longiflocosa* (Diptera: Psychodidae) posible vector en el foco de leishmaniasis cutánea del municipio de Planadas, zona cafetera del Tolima. Biomedica 1999 19(3), 239–244.

[18] Galati EAB. Apostila de Bioecologia e Identificação de Phlebotominae (Diptera, Psychodidae)–Departamento de Epidemiologia, Faculdade de Saúde Pública da USP, São Paulo, Brasil, 2015, 127pp. Available: http://www.fsp.usp.br/~egalati.

[19] Barreto P. Artrópodos hematófagos del río Raposo, Valle, Colombia IV. Psychodidae. Caldasia 1969;10(49):459–472

[20] Osorno ME, Morales AA, De Osorno F, Ferro C. Phlebotominae de Colombia (Diptera, Psychodidae) IX. Distribución geográfica de especies de Brumptomyia França & Parrot, 1921 y Lutzomyia França, 1924 encontradas en Colombia, S. A. Rev Acad Colomb Cienc Exact Fis Nat. 1972;14:(53):45–68.

[21] Young D. A review of the bloodsucking Psychodidae of Colombia (Diptera: Phlebotominae and Sycoracinae). Gainesville, Florida: University of Florida; 1979 p. 109–111, 155–157.

[22] Young D, Duncan A. Guide to the identification and geographic distribution of *Lutzomyia* sand flies in Mexico, the West Indies, Central and South America (Diptera: Psychodidae) Gainesville. Florida 32614–0103. U.S.A: Taylor and Francis; 1994 p. 341–359, 444–469.

[23] Sandoval CM, Gutiérrez R, Cárdenas R, Ferro C. Species of *Lutzomyia* (Psychodidae, Phlebotominae) in endemic cutaneous and visceral leishmaniasis foci of the department of Santander, in the eastern range of the Colombian Andes.Biomedica. 2006 10;26 Suppl 1:218–27. 17361857

[24] Hijmans RJ, Cameron SE, Parra JL, Jones PG, Jarvis A. Very high resolution interpolated climate surfaces for global land areas. Int J Climatol. 2005 25(15), 1965–1978.

[25] SIVIGILA Vigilancia rutinaria por eventos municipal y departamental.Instituto Nacional De Salud. 2014. Available: http://www.ins.gov.co/lineas-de-accion/Subdireccion-Vigilancia/sivigila/Paginas/vigilancia-rutinaria.aspx.

[26] DANE. Estimación y proyección de población nacional, departamental y municipal por sexo, grupos quinquenales de edad y edades simples de 0 a 26 años 1985–2020. 2014. Available: http://www.dane.gov.co/index.php/poblacion-y-demografia/proyecciones-de-poblacion.

[27] Etter A. Mapa general de Ecosistemas de Colombia (Escala 1:2 000 000). Instituto Alexander von Humboldt y PNUD, Bogotá 1998.

[28] de Almeida PS, de Andrade AJ, Sciamarelli A, Raizer J, Menegatti JA, Hermes SC, et al Geographic distribution of phlebotomine sandfly species (Diptera: Psychodidae) in Central-West Brazil. Mem Inst Oswaldo Cruz. 2015 6;110(4):551–9. 10.1590/0074-02760140462 26018450 PMC4501420

[29] Pardo RH, Cabrera OL, Becerra J, Fuya P, Ferro C. *Lutzomyia longiflocosa* as suspected vector of cutaneous leishmaniasis in a focus of cutaneous leishmaniasis on the sub-andean region of Tolima department, Colombia, and the knowledge on sandflies by the inhabitants. Biomedica. 2006 10;26 Suppl 1:95–108. 17361846

[30] Agudelo LA, Uribe J, Sierra D, Ruiz F, Velez ID. Presence of American cutaneous leishmaniasis vectors surrounding the city of Medellín, Colombia. Mem Inst Oswaldo Cruz. 2002 7;97(5):641–2. 12219126

[31] Santamaría E, Ponce N, Zipa Y, Ferro C. Presence of infected vectors of *Leishmania* (V.) *panamensis* within dwellings in two endemic foci in the foothill of the middle Magdalena valley, western Boyacá, Colombia. Biomedica. 2006 10; 26 Suppl 1:82–94. 17361845

[32] Montoya-Lerma J, Cadena H, Segura I, Travi BL. Association of *Lutzomyia columbiana* (Diptera: Psychodidae) with a leishmaniasis focus in Colombia due to species of the *Leishmania mexicana* complex. Mem Inst Oswaldo Cruz. 1999 May-Jun;94(3):277–83. 10348975 10.1590/s0074-02761999000300001

[33] Ovallos FG, Silva YR, Fernandez N, Gutierrez R, Galati EA, Sandoval CM. The sandfly fauna, anthropophily and the seasonal activities of *Pintomyia spinicrassa* (Diptera: Psychodidae: Phlebotominae) in a focus of cutaneous leishmaniasis in northeastern Colombia. Mem Inst Oswaldo Cruz. 2013 5;108(3). 10.1590/S0074-02762013000300007 23778653 PMC4005568

[34] Zuluaga WA, López YL, Osorio L, Salazar LF, González MC, Ríos CM, et al Twenty-year surveillance of insects relevant to public health during the construction of hydroelectric facilities in Antioquia, Colombia. Biomedica. 2012 Sep;32(3):321–31. 10.1590/S0120-41572012000300003 23715181

[35] Rotureau B, Gaborit P, Issaly J, Carinci R, Fouque F, Carme B. Diversity and ecology of sand flies (Diptera: Psychodidae: Phlebotominae) in coastal French Guiana. Am J Trop Med Hyg. 2006 7;75(1):62–9. 16837710

[36] Valderrama A, Tavares MG, Dilermando Andrade Filho J. Phylogeography of the *Lutzomyia gomezi* (Diptera: Phlebotominae) on the Panama Isthmus. Parasit Vectors. 2014 1 8;7:9 10.1186/1756-3305-7-9 24398187 PMC3892078

[37] Muñoz G, Davies CR. *Leishmania panamensis* transmission in the domestic environment: the results of a prospective epidemiological survey in Santander, Colombia. Biomedica. 2006 10;26 Suppl 1:131–44. 17361849

