# Supplementary material for: Spatial Distribution of Sand Fly Vectors and Eco-Epidemiology of Cutaneous Leishmaniasis Transmission in Colombia
Source: PLoS One. 2015 Oct 2;10(10):e0139391. doi: 10.1371/journal.pone.0139391 (PMC4592259; doi:10.1371/journal.pone.0139391)

S1 Fig. Spatial distribution of sandfly species of medical importance by genus. The Andean Mountains are shown a) *Pintomyia* b) *Psychodopygus* c) *Lutzomyia* d) *Nyssomyia* e) *Bichromomyia* and *Psathyromyia* genus.

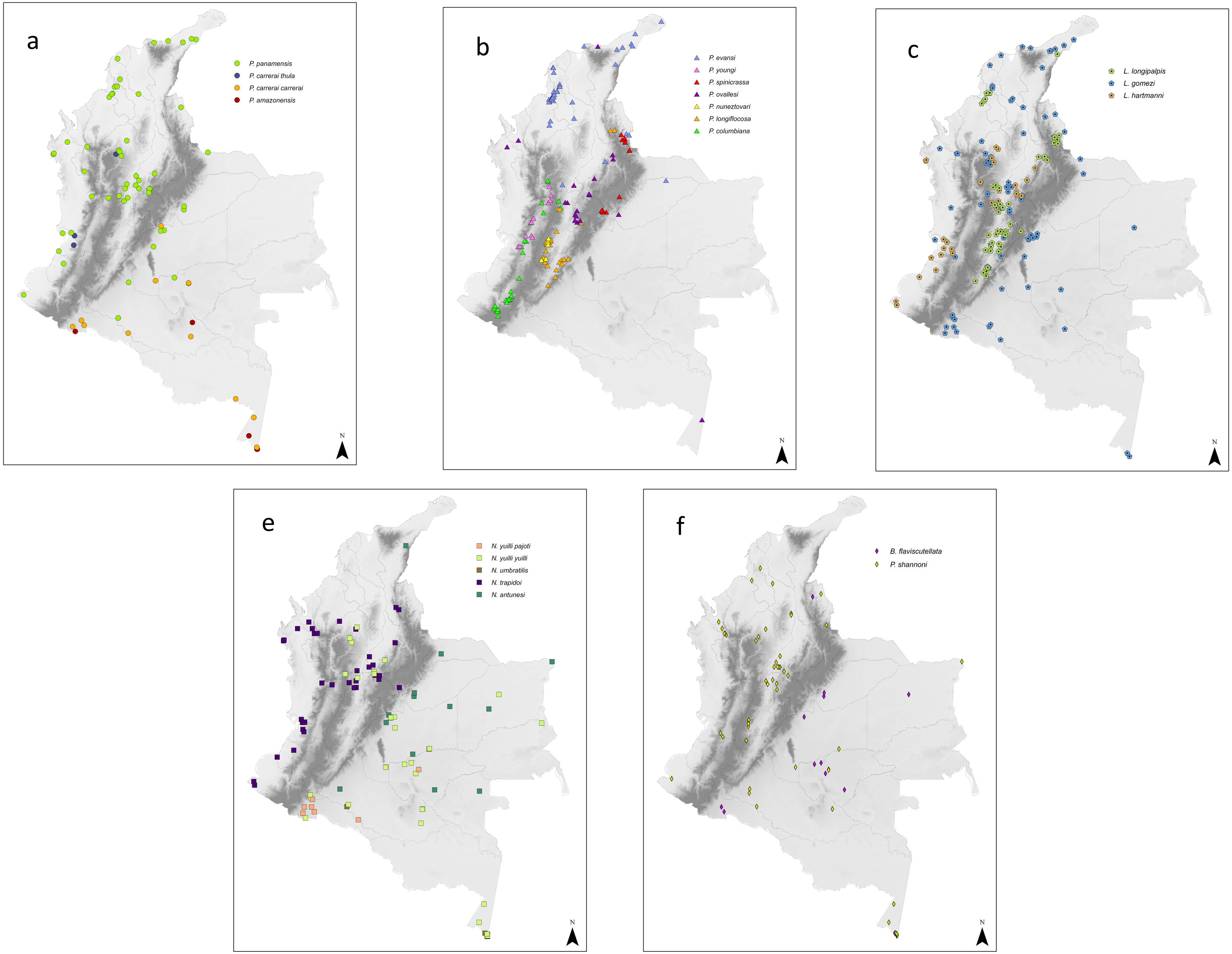

Supplement: S1 Fig — The Andean Mountains are shown a) Pintomyia b) Psychodopygus c) Lutzomyia d) Nyssomyia e) Bichromomyia and Psathyromyia genus. (PDF) [file pone.0139391.s001.pdf]
