# Supplementary material for: Spatial Distribution of Sand Fly Vectors and Eco-Epidemiology of Cutaneous Leishmaniasis Transmission in Colombia
Source: PLoS One. 2015 Oct 2;10(10):e0139391. doi: 10.1371/journal.pone.0139391 (PMC4592259; doi:10.1371/journal.pone.0139391)
Supplement: S1 Table — (PDF) [file pone.0139391.s002.pdf]

S1 Table. List and incrimination status of all species included in the study.

Genus	Species	Infected with	Country	Status<br>S (suspected)<br>P (proven)	Reference
Bichromomyia	*flaviscutellata*	*Leishmania complejo mexicana*	Colombia	P	Montoya-Lerma *et al.,* 1999
		Leishmania amazonensis	Colombia, Brazil,French Guyana, Venezuela, Ecuador	P	Rotureau *et al.,*2006; Souza *et al.,* 1996; Grimaldi *et al.,* 1989; Young & Lawyer, 1987; Arias *et al.,*1987.
Lutzomyia	*gomezi*	*Leishmania panamensis*	Panama, Colombia	P	Valderrama et al., 2014; Echeverry *et al.,* 2005; Christensen *et al.,*1983
		Leishmania braziliensis	Venezuela	P	Ponce *et al.,* 2006; Santamaria et al.,2006; Travi et al., 1988; Gomez & Hashiguchi 1987; Jhonson et al.,1963.
Lutzomyia	*longipalpis*	*Leishmania braziliensis, Leishmania amazonensis, Leishmania mexicana*	Brazil	S	Savani *et al.,* 2009 ; Lopez *et al.,* 1996.
Lutzomyia	*hartmanni*	*Leishmania panamensis*	Ecuador	S	Grimaldi et al 1989; (CIPA GROUP)
		Leishmania colombiensis	Colombia	P	Perez-Doria et al., 2011; Ponce et al.,2006; Pardo et al.,2006; Davies et al.,2000; Kreutzer *et al.,*1991.
Nyssomyia	*antunesi*	*Leishmania spp.*	Colombia	S	Adolfo Vásquez-Trujillo *et al.*, 2013; Cabrera-quintero OL *et al.*, 2008
Nyssomyia	*trapidoi*	*Leishmania panamensis*	Colombia, Ecuador, Costa Rica, Honduras, Panama	P	Ponce et al., 2006; Pardo et al., 2006; Echeverry et al., 2005; Davies et al., 2000; Grimaldi et al., 1989; Murillo & Zeledon, 1985; Young & Lawyer, 1987.
Nyssomyia	*yuilli**	*Leishmania panamensis*	Colombia	S	Ponce *et al.,*2006
		Leishmania guyanensis	Brasil	S	Ready *et al.,* 1986
Nyssomyia	*umbratilis*	*Leishmania guyanensis*	Colombia, French Guyana, Brazil	P	Rotureau et al., 2006; Echeverry et al., 2005; Davies et al., 2000; Young & Duncan 1994; Young & Lawyer 1987; Christensen *et al.,* 1983;
Pintomyia	*evansi*	*Leishmania mexicana,*	Colombia	S	Bejarano et al., 2002; Vivenes *et al.,* 2001
		Leishmania amazonensis	Colombia	S	Bejarano et al., 2002; Vivenes et al., 2001

*(Reference only available for species not for subspecies)

Genus	Species	Infected with	Country	Status S (suspected) P (proven)	Reference
Pintomyia	*columbiana*	*Leishmania braziliensis*	Colombia	S	Pardo *et al*., 2006; Bejarano E *et al*.,2003; Montoya-lerma *et al*.,1999. (CIPA GROUP)
		Leishmania mexicana	Colombia	S	Pardo *et al*., 2006; Bejarano E *et al*., 2002; Montoya-Lerma *et al*.,1999. (CIPA GROUP)
		Leishmania panamensis	Colombia	S	Pardo *et al*., 2006; Bejarano E *et al*., 2002; Montoya-Lerma *et al*.,1999. (CIPA GROUP)
Pintomyia	*longiflocosa*	*Leishmania (V) braziliensis*	Colombia	P	Pardo *et al*.,2006; Echeverry *et al*., 2005; Ferro et al.,1997.
		Leishmania (Vianna) guyanensis	Colombia	S	Ferro *et al*,. 2011
		Leishmania sp.	Bolivia	S	Young & Duncan 1994
		Leishmania braziliensis	Colombia	S	Pardo *et al*., 2006.
Pintomyia	*ovallesi*	*Leishmania braziliensis*	Colombia, Venezuela, Guatemala	P	Feliciangeli 1991; Rowton *et al*.,1992; Davies *et al*., 2000; Bejarano *et al*., 2003; Echeverry *et al*., 2005; Davies *et al*., 2000
		Leishmania mexicana	Venezuela	P	Davies *et al*., 2000; Young & Duncan 1994
Pintomyia	*spinicrassa*	*Leishmania braziliensis*	Colombia, Venezuela	P	Feliciangeli, 1991; Young & Duncan 1994
Pintomyia	*youngi*	*Leishmania panamensis*	Costa Rica	S	Young & Duncan 1994
		Leishmania braziliensis	Colombia, Venezuela	S	Echeverry, 2005; Bejarano *et al*., 2002; Davies et al., 2000.
Pintomyia	*nuñeztovari*	*Leishmania amazonensis*	Bolivia	S	Torres et al. 1989; Torres et al. 1998; Martinez et al. 1999;
		Leishmania braziliensis	Bolivia	S	Le Pont & Desjeux 1984
Psathyromyia	*shannoni*	*Leishmania braziliensis*	Bolivia	S	Young & Duncan 1994
		Leishmania sp	Colombia, Guatemala, Belice	S	Young & Duncan 1994 (CIPA GROUP)
Psychodopygus	*amazonensis*	*Leishmania naiffi*	Brazil	S	M. Maroli *et al*., 2013
Psychodopygus	*carrerai**	*Leishmania braziliensis*	Brazil	S	Grimaldi *et al*., 1989 (CIPA GROUP)
Psychodopygus	*panamensis*	*Leishmania panamensis*	Colombia	S	Echeverry *et al*., 2005.
		Leishmania panamensis	Panama	P	Kreutzer *et al*., 1991; Grimaldi *et al*., 1989.
		Leishmania colombiensis	Panama	P	Kreutzer *et al*,. 1991; Grimaldi *et al*., 1989.
		Leishmania braziliensis	Venezuela, Guatemala	P	Young & Duncan 1994 ; Feliciangeli, 1991.

*(Reference only available for species not for subspecies)

References in Supporting Information Table 1

Arias JR, Freitas RA, Naiff RD, Barrett TV. Observations on the parasite Leishmania mexicana amazonensis and its natural infection of the sand fly Lutzomyia olmeca nociva. Bull PAHO. 1987. 21: 48-53.

Bejarano E , Uribe S, Rojas W, & Vélez ID. Phlebotomine sand flies (Diptera: Psychodidae) associated with the appearance of urban leishmaniasis in the City of Sincelejo, Colombia. Memorias Do Instituto Oswaldo Cruz, 2002 97(July), 645–647.

Cabrera-quintero O et al. Lutzomyia antunesi ,probable vector de leishmaniasis cutanea en el area rural de villavicencio., Rev. Salud Publica. 2008. 10(4), 625–632.

Christensen H, Fairchild GB, Herrer A, Johnson C, Young D, de Vásquez A. The ecology of cutaneous, leishmaniasis in the Republic of Panama. J Med Entomol 1983. 20: 463-484.

Davies C, Reithinger R, Campbell-Lendrum D, Feliciangeli D, Borges R, Rodriguez N. The epidemiology and control of leishmaniasis in Andean countries. Cad. Saúde Pública 2000 16(4): 925-950.

Echeverry MC, Milena S, & Trujillo G. Guía de atención de la leishmaniasis. Ministerio de La Proteccion Social. Programa de Apoyo a La Reforma de Salud/PARS. 2005. 21, 176–219.

Feliciangeli MD. Vectors of leishmaniasis in Venezuela. Parassitologia 1991. 33: 229-236

Ferro C, Marín D, Góngora R, Carrasquilla M, Trujillo J, Rueda N, Ocampo C. Phlebotomine vector ecology in the domestic transmission of American cutaneous leishmaniasis in Chaparral, Colombia. The American Journal of Tropical Medicine and Hygiene, 2011 85(5), 847–56.

Ferro C, Pardo R, Torres M, Morrison A. Larval microhabitats of Lutzomyia longipalpis (Diptera: Psychodidae) in an endemic focus of visceral leishmaniasis in Colombia. Journal of Medical Entomology 1997. 34: 719-728.

Gomez E, Hashiguchi Y. Natural infection of sand flies with Leishmania promastigotes. InY Hashiguchi,Studies on New World Leishmaniasis and its Transmission, with Particular Reference to Ecuador, Kyowa Printing & Co, Kochi, Japan, 1987. p. 70-78.

Grimaldi G, Tesh R, McMahon-Pratt D. A review of geographic distribution and epidemiology of leishmaniasis in the new world. Am J Trop Med Hyg 1989 41: 687-725.

Johnson PT, McConnell E, Hertig M. Natural and experimental infections of Leptomonad flagellates in Panama Phlebotomus sandflies. Exp Parasitol.1963.14: 107-122.

Kreutzer RD, Corredor A, Grimaldi G Jr, Grogl M, Rowton ED, Young DG, Morales A, McMahon-Pratt D, Guzman H, Tesh RB. Characterization of Leishmania colombiensis sp. n (Kinetoplastida: Trypanosomatidae), a new parasite infecting humans, animals, and phlebotomine sand flies in Colombia and Panama. Am J Trop Med Hyg. 1991. 44: 662-75.

Lawyer PG, and Young DG. Experimental transmission of Leishmania mexicana 10 hamslers by biles of Phlebotomine sand flies (Diplera: Psychodidae) from the Uniled Slales. J. Med. Entomol. 1987. 24: 458-462.

Le Pont, F & Desjeux, P. Leishmaniasis in Bolivia-VI. Observations on Lutzomyia nuneztovari Anglesi Le Pont & Desjeux, 1984 The presumed vector of tegumentary leishmaniasis in the Yungas focus. Rio de Janeiro: 1984. Mem. Inst. Oswaldo Cruz.

Lopez Y, Osorio L, Alvarez G, Rojas J, Jimenez F, Gomez C. Sandfly Lutzomyia longipalpis in a cutaneous leishmaniasis focus in central Colombia. Memórias do Instituto Oswaldo Cruz .1996. 91: 415-419.

Maroli M, Feliciangeli MD, Bichaud L, Charrel RN, Gradoni L Phlebotominae sandflies and the spreading of leishmaniases and other diseases of public health concern. Med Vet Entomol 2013. 27: 123-147

Martinez E, Le Pont F, Torres M, Telleria J, Vargas F, Dujardin JC et al. Lutzomyia nuñeztovari anglesi (Le Pont & Desjeux, 1984) as a vector of Leishmania amazonensis in a sub-Andean leishmaniasis focus of Bolivia. Am J Trop Med Hyg 1999;61:846-9.

Montoya-Lerma J, Cadena H, Segura I, & Travi B L. Association of Lutzomyia columbiana (Diptera: Psychodidae) with a leishmaniasis focus in Colombia due to species of the Leishmania mexicana complex. Memórias Do Instituto Oswaldo Cruz, 1999 94(3), 277–83.

Pardo R, Cabrera O, Becerra J, Fuya P, Ferro C. Lutzomyia longiflocosa, posible vector en un foco de leishmaniasis cutánea en la región subandina del departamento del Tolima, Colombia, y el conocimiento que tiene la población sobre este insecto Biomédica 2006, 26

Pérez-Doria A, Patermina L, Patermina M, Martínez L, Vervel_Vergara D, Bejarano EE- Infección natural de Lutzomyia evansi con especies del complejo Leishmania braziliensis causantes de leishmaniasis cutánea en la costa Caribe Colombiana. Biomédica, 2011. 31

Ponce N, Zipa Y, & Ferro C. Presencia en el peridomicilio de vectores infectados con Leishmania (Viannia) panamensis en dos focos endémicos en el occidente de Boyacá , piedemonte del valle del Magdalena medio , Colombia, Biomédica. 2006. 26, 82–94.

Ready P, Arias J, Freitas R. 1985. A pilot study to control Lutzomyia umbrataiis (Diptera: Psychodidae), the major vector of Leishmania braziliensís guyanensis, Amazonas in a peri-urban rainforest of Manaus,State, Brazil. Mem. Inst. Oswaldo. Cruz. 80 (1): 27-36.

Rotureau, B, Gaborit, P, Issaly, J, Carinci, R, Fouque, F, & Carme, B. Diversity and ecology of sand flies (Diptera: Psychodidae: Phlebotominae) in coastal French Guiana. The American Journal of Tropical Medicine and Hygiene, 2006 75(1), 62–9.

Santamaría E, Ponce N, Zipa Y, Ferro C. Presencia en el peridomicilio de vectores infectados con Leishmania (Viannia) panamensis en dos focos endémicos en el occidente de Boyacá, piedemonte del valle del Magdalena medio, Colombia. Biomédica. 2006. 26(Supl.1):82-94.

Savani ES, Nunes VL, Galati EA, Castilho TM, Zampieri RA, Floeter-Winter LM. The finding of Lutzomyia almerioi and Lutzomyia longipalpis naturally infected by Leishmania spp in a cutaneous and canine visceral leishmaniases focus in Serra da Bodoquena, Brazil. Vet Parasitol 2009. 160: 18-24

Souza A, Ishikawa E, Braga R, Silveira F, Lainson R, and Shaw, J. Psychodopygus complexus, a new vector of Leishmania braziliensis to humans in Pará State, Brazil. Trans. R. Soc. Trop. Med. 1996 Hyg. 90: 112–113.

Torres M, Lopez M, Le Pont F, Martinez E, Munoz M, Hervas D, et al. Lutzomyia nuneztovari anglesi (Diptera: Psychodidae) as a probable vector of Leishmania braziliensis in the Yungas, Bolivia. Acta Trop 1998;71:311-6.

Torres JM, Le Pont F, Mouchet J, Desjeux P, Richard A. Epidemiologie de la leishmaniose tegumentaire en Bolivie. 1. Description des zones d’etude et frequence de la maladie. Ann Soc Belge Med Trop 1989;69:297-306.

Travi BL, Montoya J, Solarte Y, Lozano L, Jaramillo C. Leishmaniasis in Colombia. I. studies on Phlebotomine fauna associated with endemic foci in the Pacific Coast region. Am J Trop Med Hyg. 1988. 39:261–266.

Trujillo A V, Reina A, Orjuela A, Suárez E, Palomares J, Stella L, & Alvarez B. Seasonal variation and natural infection of Lutzomyia antunesi (Diptera : Psychodidae : Phlebotominae), an endemic species in the Orinoquia region of Colombia. Mem Inst Oswaldo Cruz, 2013.108, 463–469.

Valderrama, A, Tavares, M G, & Filho, J D A. Phylogeography of the Lutzomyia gomezi (Diptera: Phlebotominae) on the Panama Isthmus. Parasites & Vectors, 2014 7(1), 9.

Vivenes A, Oviedo M, Márquez JC, & Montoya-Lerma J. Effect of a Second Bloodmeal on the Oesophagus Colonization by Leishmania mexicana Complex in Lutzomyia evansi (Diptera: Psychodidae). Memorias Do Instituto Oswaldo Cruz, 2001. 96, 281–283.

Young DG, Duncan MA. Guide to the identification and geographic distribution of Lutzomyia sand flies in Mexico, the West Indies, Central and South America (Diptera: Psychodidae). Mem Amer Entomol Inst. 1994.54: 1-881.
