# Supplementary material for: Spatial Distribution of Sand Fly Vectors and Eco-Epidemiology of Cutaneous Leishmaniasis Transmission in Colombia
Source: PLoS One. 2015 Oct 2;10(10):e0139391. doi: 10.1371/journal.pone.0139391 (PMC4592259; doi:10.1371/journal.pone.0139391)
Supplement: S2 Table — (PDF) [file pone.0139391.s003.pdf]

S2 Table. Percent of area covered by potential distribution of each sand fly species in areas of cutaneous leishmaniasis by prevalence.

**Species / Prevalence**	***P. shannoni* (%)**	***P. panamensis* (%)**	***P. ovallesi* (%)**	***P. nuneztovari* (%)**	***P. longiflocosa*(%)**	***P. columbiana* (%)**	***P. amazonensis* (%)**	***N. yuilli pajoti* (%)**	***N. umbratilis* (%)**	***N. trapidoi* (%)**	***L. antunesi* (%)**	***L. longipalpis* (%)**	***L. hartmanni* (%)**	***L. gomezi* (%)**	***L. evansi* (%)**	***P. youngi* (%)**	***P. spinicrassa* (%)**
0.33 to 101.35	38.06	45.22	49.84	46.69	49.38	54.20	35.64	36.67	37.73	39.98	27.02	54.65	44.46	44.90	68.56	50.85	45.91
102.53 to 405.26	22.77	18.85	13.43	10.31	12.71	8.77	19.58	18.54	16.00	17.53	37.42	11.13	14.81	16.00	5.05	5.97	8.05
423.86 to 1071.31	11.81	9.74	9.92	5.04	3.75	3.53	12.06	12.25	13.37	20.61	13.70	7.29	16.45	8.86	3.00	3.14	2.96
1071.31 to 2275.56	2.02	2.16	3.54	2.89	4.36	1.25	0.89	0.75	1.10	4.19	0.82	2.34	3.35	2.40	0.01	0.86	0.64
2275.56 to 3643.82	2.78	2.87	1.12	1.09	1.80	1.37	3.40	3.36	2.87	1.76	5.25	0.06	1.93	3.32	0.00	0.54	0.00
No Data	22.56	21.15	22.16	33.98	28.00	30.88	28.43	28.44	28.93	15.92	15.79	24.53	19.00	24.53	23.39	38.64	42.44
